# A Density Functional Tight Binding Study of Acetic Acid Adsorption on Crystalline and Amorphous Surfaces of Titania

**DOI:** 10.3390/molecules20023371

**Published:** 2015-02-17

**Authors:** Sergei Manzhos, Giacomo Giorgi, Koichi Yamashita

**Affiliations:** 1 Department of Mechanical Engineering, National University of Singapore, Block EA #07-08, 9 Engineering Drive 1, Singapore 117576, Singapore; 2 Department of Chemical System Engineering, School of Engineering, University of Tokyo, 7-3-1, Hongo, Bunkyo-ku, Tokyo 113-8656, Japan; E-Mails: giacomo@tcl.t.u-tokyo.ac.jp (G.G.); yamasita@chemsys.t.u-tokyo.ac.jp (K.Y.); 3 CREST-JST, 7 Gobancho, Chiyoda-ku, Tokyo 102-0076, Japan

**Keywords:** titanium dioxide, anatase, rutile, (B)-TiO_2_, amorphous TiO_2_, acetic acid, adsorption, dye-sensitized solar cells, density functional tight binding

## Abstract

We present a comparative density functional tight binding study of an organic molecule attachment to TiO_2_ via a carboxylic group, with the example of acetic acid. For the first time, binding to low-energy surfaces of crystalline anatase (101), rutile (110) and (B)-TiO_2_ (001), as well as to the surface of amorphous (*a*-) TiO_2_ is compared with the same computational setup. On all surfaces, bidentate configurations are identified as providing the strongest adsorption energy, *E_ads_* = −1.93, −2.49 and −1.09 eV for anatase, rutile and (B)-TiO_2_, respectively. For monodentate configurations, the strongest *E_ads_* = −1.06, −1.11 and −0.86 eV for anatase, rutile and (B)-TiO_2_, respectively. Multiple monodentate and bidentate configurations are identified on *a*-TiO_2_ with a distribution of adsorption energies and with the lowest energy configuration having stronger bonding than that of the crystalline counterparts, with *E_ads_* up to −4.92 eV for bidentate and −1.83 eV for monodentate adsorption. Amorphous TiO_2_ can therefore be used to achieve strong anchoring of organic molecules, such as dyes, that bind via a -COOH group. While the presence of the surface leads to a contraction of the band gap *vs.* the bulk, molecular adsorption caused no appreciable effect on the band structure around the gap in any of the systems.

## 1. Introduction

Titanium oxide is widely used in various electrochemical technologies, namely heterogeneous catalysis and photocatalysis [1,2], fuel cells [3,4], solar cells (dye-sensitized, DSSC [5,6,7,8,9], and perovskite-sensitized, PSSC [10,11]) and electrochemical batteries [12,13,14,15,16,17,18]. It is relatively abundant, inexpensive, safe and possesses a band structure suitable for these electrochemical and photocatalytic applications [19]. The three most stable phases are usually employed: anatase, rutile and (B). In all of these applications, molecules interact with titania surfaces. While some higher-energy, higher-index surfaces are sometimes explored [20,21], most applications have relied on low-energy anatase (101), rutile (110) and (B) (001) surfaces [1,2,22,23,24], which is justified by the ease of synthesis and stability.

In the applications in DSSC anodes, as well as in studies of carboxylic acids on TiO_2_, molecules typically bind to TiO_2_ via the -COOH group, with bidentate bridging and monodentate configurations dominating [9,25,26,27,28,29,30]. Bidentate chelating configurations are sometimes considered, but are usually much less stable [9,25,31,32]. Adsorption of organic molecules via a carboxylic moiety is also important for other applications. For example, the 21 amino acids possess the -COOH group, which plays a role in adsorption on TiO_2_. These interactions are of interest for the development of biocompatible and bio-inspired nanostructured materials [33]. A key parameter of the interaction of molecules with TiO_2_ surfaces is binding strength. It controls the stability of DSSC anodes, as well as the degree of electronic coupling, which directly influences the electron transfer rate [26,34]. It also controls the reaction rates in catalytic applications [1,2]. The ability to control or to strengthen binding by the choice of a specific TiO_2_ surface is therefore important in all of these applications.

Applications of these crystalline phases and surfaces have also been supported by extensive theoretical and computational studies [35,36,37,38,39] and, specifically, studies of molecular adsorption via the carboxylic group onto selected crystalline phases [9,25,26,27,28,29,31,32]. Amorphous titania (*a*-TiO_2_) has recently attracted interest for its use in the dye-sensitized technology [40,41,42], as well as in electrochemical batteries [43,44,45,46,47,48] and in photocatalysis [49]. These studies indicate that *a*-TiO_2_ may be advantageous in these applications for several reasons. For example, the rate capability of battery electrodes made of nanostructured amorphous TiO_2_ was found to be higher than that of nanostructured anatase TiO_2_ due to the higher Li-diffusion coefficient in amorphous TiO_2_ [44,47]. *a*-TiO_2_ has also been shown to possess a distribution of binding energies to Li, Na and Mg, with the lowest binding energy stronger than that of the anatase, rutile and (B) phases [50,51]. The same might hold for molecular adsorption and needs to be quantified. The presence of amorphous TiO_2_ in the anodes of DSSC has been shown to improve conversion efficiency [42]. Low-crystallinity TiO_2_ DSSC anodes have shown a high open circuit voltage and low recombination [41]. *a*-TiO_2_ could also be a cheaper alternative to crystalline phases for photocatalytic applications, as it possesses similar band gap characteristics to anatase [49,52].

There are still few computational studies of the properties of *a*-TiO_2_ relevant for these applications [40,49,50,51,52,53]. Specifically, there are no *ab initio* studies comparing at the same level of theory and with the same computational parameters the adsorption of organic molecules on the surfaces of all of the phases of TiO_2_ that are finding use in electrochemical technologies, *i.e.*, anatase, rutile, (B) and amorphous. As *ab initio* methods still possess only semi-quantitative accuracy, results obtained with different phases in different studies using different computational setups are not directly comparable. Here, we present a study that compares with the same computational setup the adsorption of a prototypical organic molecule endowed with a -COOH anchoring group (acetic acid) to low energy crystalline surfaces, namely, anatase (101), rutile (110) and (B) (001), and to amorphous surfaces. We therefore aim to compare for the first time the adsorption modes among different phases and to understand the effect of amorphization on the geometries and binding strength of molecules.

## 2. Theoretical and Computational Methods

Calculations were performed using the self-consistent charge density functional tight binding scheme (SCC-DFTB) [54] and the DFTB + code [55]. SCC-DFTB is an approximate DFT approach derived from a second-order expansion of the DFT energy with respect to charge density fluctuations and provides *ab initio* accuracy for systems for which it is parameterized. We used the parameter set (Slater–Koster files) “matsci-0-3”, which has been benchmarked, in particular, for TiO_2_ and for organic molecules interacting with TiO_2_ [56].

For bulk TiO_2_ calculations, we used conventional standard cells with 4, 2 and 8 formula units for anatase, rutile and (B), respectively. The Brillouin zone was sampled with a *k*-point density no less than one point per 30^−1^ Å^−1^. Converged results were obtained with 10 × 10 × 5, 8 × 8 × 12, and 3 × 10 × 6 *k*-points for anatase, rutile and (B), respectively. The initial structure of *a*-TiO_2_ was taken from [52] and re-optimized within the present setup. A 192-atom supercell of a size of about 13 × 13 × 13 Å was used, and converged results were obtained with 3 × 3 × 3 *k*-points.

Anatase (101), rutile (110) and B (001) low-energy surfaces were modeled with slabs of a thickness of at least 13 Å, which are shown in Figure 2, Figure 3 and Figure 4. The lateral (*xy*) extent of the supercell was about 10.5 × 11.5 Å, 12 × 13 Å and 12.5 × 11.3 Å, for anatase, rutile and (B), respectively. The top half of the slab (in the *z* direction) was optimized, and the bottom half held at bulk positions during optimization. The amorphous surfaces were obtained from the bulk supercell by adding a vacuum in the *z* direction. Two surfaces were obtained by optimizing the top/bottom half and fixing the bottom/top half of the slab; these are labeled as “top” and “bottom” surfaces. This is a similar approach to that used in [53]. All surface supercells were 30 Å in the *z* direction, and 3 × 3 × 1 *k*-points were used. Acetic acid molecules were then positioned above the surfaces in various configurations with the COOH group in monodentate or bidentate binding to the surface and optimized with DFTB. We here focus on bidentate binding in bridging configurations, which are known to be much more stable than chelating configurations [9,25,31,32].

The adsorption energy (*E_ads_*) of AcOH on top of all of the surfaces here considered has been calculated as:

(1) $$E_{ads}=E_{surf\;+\;AcOH}-\left(E_{surf}+E_{AcOH}\right)$$

where *E_surf_*, *E_AcOH_* and *E_surf + AcOH_* represent the energy of, respectively, the optimized surface, a free optimized acetic acid molecule and of the final optimized anchored system formed by the surface and acetic acid.

## 3. Results and Discussion

### 3.1. TiO_2_ Bulk and Surfaces

**Figure 1:**
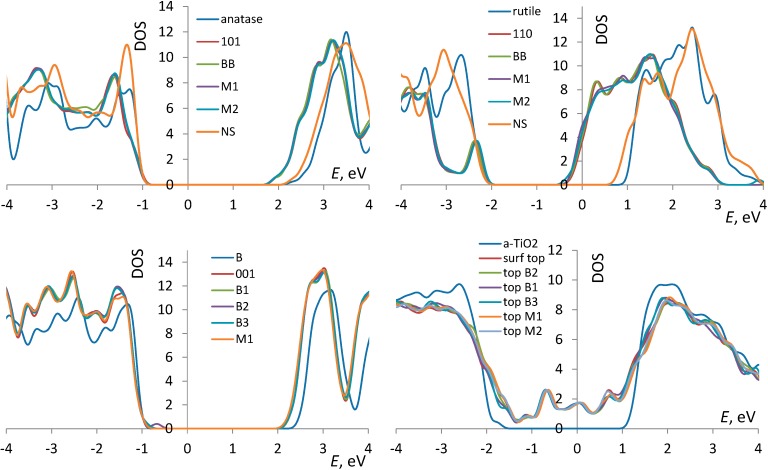
The densities of states (DOSs) of bulk anatase, rutile, (B) and *a*-TiO_2_ and their surfaces, as well as surfaces with AcOH adsorbed in different configurations (described in Section 3.1). For each phase, the zero of the energy axis corresponds to the Fermi energy in the bulk, and the DOSs of surfaces are plotted to match the valence band maximum. Gaussian broadening of 0.1 eV. For *a*-TiO_2_, only the “top” surface data are shown. The curves for the “bottom” surface are qualitatively similar. For (B)-TiO_2_ and *a*-TiO_2_, curves for the selected adsorption configurations are shown; the curves for other adsorption configurations are similar. For anatase and rutile, the DOSs for nanosheets (“NS”) are also shown.

Anatase: The cell vectors of the optimized bulk anatase (conventional standard cell) are *a* = *b* = 3.81 Å, *c* = 9.73 Å, in good agreement with previously reported experimental and computed values [57,58,59,60,61]. The energy per formula unit is −215.181 eV. The density of states is shown in Figure 1 and shows a band gap of about 3.2 eV, in good agreement with experimental values of 3.2–3.3 eV [62] and prior DFTB calculations [63]. The presence of the (101) surface causes a contraction of the band gap by about 0.5 eV.

**Figure 2:**
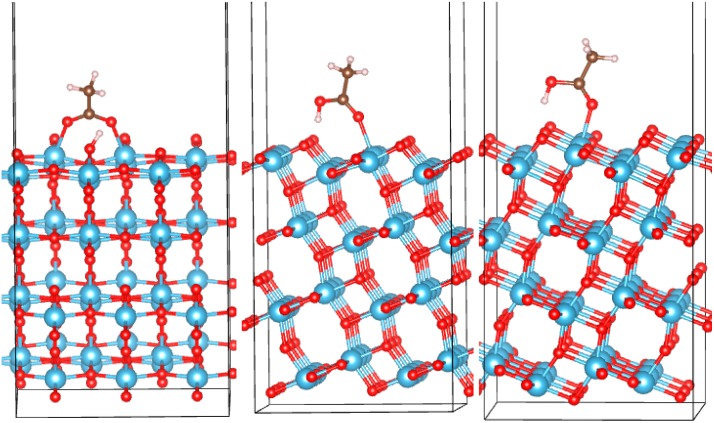
Adsorption configurations of AcOH on the anatase (101) surface of TiO_2_: bidentate (BB) (left), M1 (monodentate) (middle) and M2 (right). The atom color code here and elsewhere: Ti, blue; O, red; C, brown; H, light grey. Visualization here and elsewhere by VESTA [87].

**Figure 3:**
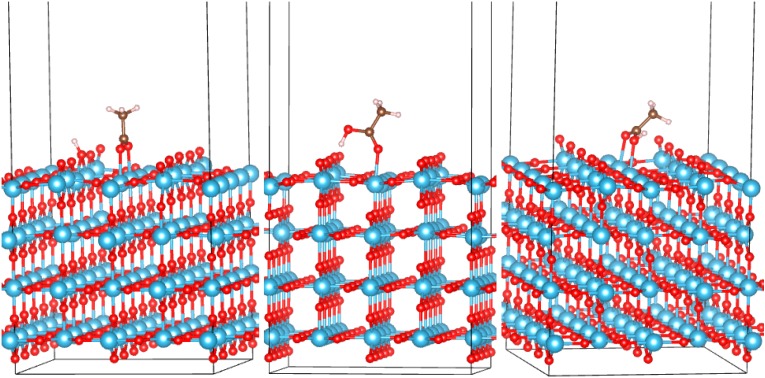
Adsorption configurations of AcOH on the rutile (110) surface of TiO_2_: BB (left), M1 (middle) and M2B (right).

**Figure 4:**
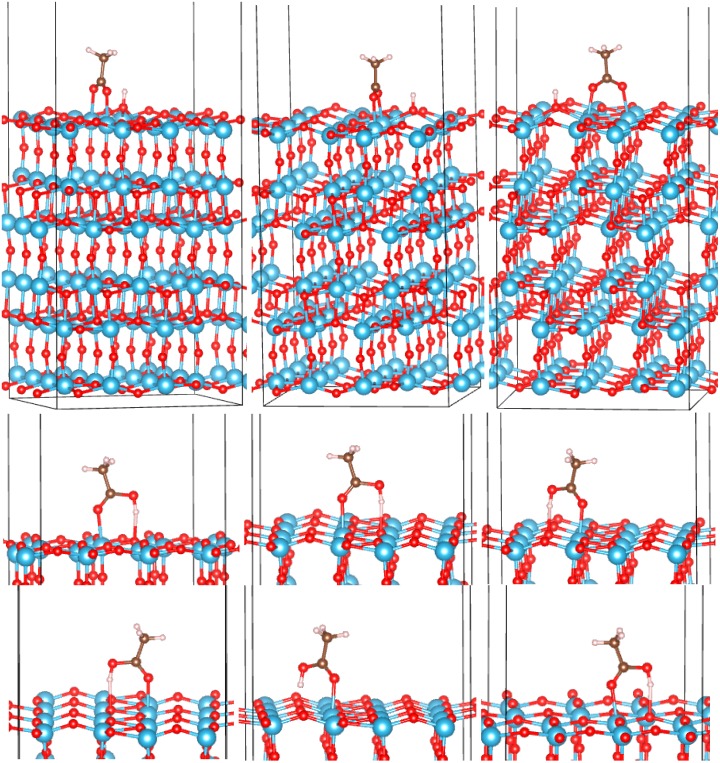
Adsorption configurations of AcOH on the (B) (001) surface of TiO_2_. Top row: BB1 to BB3 (left to right). Bottom rows: M1 to M6 (left to right and top to bottom).

Rutile: The cell vectors of the optimized rutile phase are *a* = *b* = 4.61 Å, *c* = 2.97 Å, in good agreement with previously reported values [57,58,61]. The density of states is shown in Figure 1 and shows a band gap of about 3.1 eV, in good agreement with reported values of about 3 eV [22,64,65,66] and consistent with that of anatase. The energy per formula unit is −215.481 eV. The calculations therefore correctly reproduce the anatase-rutile phase ordering, albeit with a difference of 0.1 eV per atom, which is higher than the reported values of 0.012–0.015 eV [67,68]. This, however, is not critical for the present purpose of studying molecular adsorption. This accuracy is also comparable with that of previously reported DFT values for cohesive energies ranging from −18.77 to −21.44 eV [69,70] for rutile and from −21.54 to −21.60 eV [70,71] for anatase. The presence of the interface ((110) surface) causes a contraction of the band gap by about 1.5 eV.

(B): The cell parameters of the optimized (B) phase are *a* = 12.45 Å, *b* = 3.76 Å, *c* = 6.69 Å, α = γ = 90°, β = 107.3°, in good agreement with previously reported values [22,72]. The density of states is also shown in Figure 1 and shows a band gap of about 3.1 eV, in good agreement with the experimental value of 3.2 eV [73]. The energy per formula unit is −215.365 eV, *i.e.*, similar to previous *ab initio* calculations, we obtain that (B) is more stable than anatase, although this is in contradiction with the low abundance of (B) relative to anatase and rutile [22,57]. The effect of the (001) surface on the band structure is the least significant among the three crystalline phases, decreasing the band gap by about 0.3 eV. This can be rationalized by the low surface energy of this surface [22], as well as by the substantial slab thickness we used (6 Ti layers *vs.* 4 Ti layers for the rutile (110) slab, which shows the strongest effect of the surface on the gap; see Figure 2, Figure 3 and Figure 4).

*a*-TiO_2_: The optimization in DFTB+ resulted in some atomic relaxation, but largely preserved the original structure of [52]. The energy per formula unit is −214.795 eV, *i.e.*, metastability by about 0.13 eV per atom *vs.* anatase, similar to that reported in [52]. The density of states shown in Figure 1 for bulk *a*-TiO_2_ shows a similar gap of about 3 eV to that of the crystalline phases, similar to the report of [52]. The introduction of the surface gives rise to multiple states in the band gap, also shown in Figure 1.

The reduction of the bandgap is partly due to the limited thickness of the slab and the fixation of the bottom layers. The reduction is much less severe if all atoms are allowed to relax (*i.e.*, a nanosheet model), as shown in Figure 1 for anatase and rutile (orange lines). We have also performed a plane wave DFT calculation on the same slab of anatase (surface and nanosheet) using the Vienna *ab initio* simulation package (VASP) code [74,75,76,77], the PBE functional [78], PAW pseudopotentials [79] and a cutoff energy of 600 eV and confirmed that the same effect is observed. Similar to DFTB results, no significant effect on the densities of state (DOS) of molecular adsorption was observed.

### 3.2. Adsorption Configurations of AcOH and Energetics

Anatase (101): Similar to previous works [31,80], a bidentate (BB) and two monodentate configurations (M1 and M2) were identified. These are shown in Figure 2, and their adsorption energies are listed in Table 1. The BB mode is therefore preferred. In the bidentate configuration, both oxygen atoms of the carboxylic group bind to surface Ti atoms with both O_mol_-Ti bonds equal to 2.22 Å. The H atom is dissociated from the molecule and is bound to a surface oxygen. In the monodentate configurations, the molecules are undissociated, with O_mol_-Ti bonds of 2.26 Å and hydrogen bonds between the H atom and a surface oxygen of 1.62 Å, in both M1 and M2 configurations. Molecular adsorption has no appreciable effect on the band structure around the gap regardless of the configuration (Figure 1).

For AcOH on anatase (101), we can compare the adsorption energies computed with DFTB (Table 1) to previous DFT calculations. Using the SIESTA code [81], the PBE functional [78], broad DZP basis functions and Troullier–Martins pseudopotentials [82], Chan *et al.* computed *E_ads_* of −1.71, −1.56 and −1.50 eV for the BB, M1 and M2 configurations, respectively, using a thinner slab than in the present study [80]. Spreafico *et al.* [30] computed *E_ads_* values of −1.25 and −1.27 eV for the BB and M1 configurations, respectively, by using the CP2K code [83], the PBE functional, Goedecker pseudopotentials [84], a mixed Gaussian-plane wave (GPW) basis [85], also employing the dispersion correction scheme of Grimme [86]. The values were also dependent on slab size [30]. In [31], the adsorption strength of the M1, M2 and BB modes was compared with different DFT setups, including a cluster of slab TiO_2_ models, localized or plane wave basis functions and GGA or hybrid functionals. The stability of M1 and M2 relative to BB was ± 0.5 eV [31]. We have also made a plane-wave DFT calculation with VASP [74,75,76,77], using the PBE functional, PAW pseudopotentials [79] and a plane wave cutoff of 600 eV. We obtained *E_ads_* values of −0.91, −0.95 and −0.91 eV for M1, M2 and BB configurations, respectively. The differences in the values of *E_ads_* of the order of half an eV observed among different commonly accepted DFT setups, including differences of the order of 0.5 eV in relative *E_ads_* for different adsorption configurations, highlight the need to use one and the same setup to obtain truly comparative values of *E_ads_* among different phases and configurations. Interestingly, a measured infrared spectrum of AcOH on the anatase (101) surface pointed to bidentate adsorption [31].

**Table 1:** Adsorption energies *E_ads_* of AcOH in different configurations on anatase (101), rutile (110) and (B) (001) surfaces of TiO_2_, as well as on two amorphous surfaces. The bond length for bonding between the molecule’s and surface atoms, as well as charges on the molecule *q* are also given. For bidentate configurations, the two bond lengths are O_mol_-Ti; for monodentate, they are O_mol_-Ti and H-O_surf_.

System	*E_ads_*, eV	*q*, |*e*|	O_mol_-Ti, Å	O_mol_-Ti/H-O_surf_, Å
Anatase (101)				
BB	−1.93	−0.12	2.22	2.22
M1	−0.93	0.16	2.26	1.62
M2	−1.06	0.17	2.26	1.62
Rutile (110)				
BB	−2.49	−0.11	2.21	2.21
M1	−1.04	0.17	2.26	1.60
M2B	−1.11	0.34	2.27	2.29
B (001)				
BB1	−0.74	−0.17	2.24	2.25
BB2	−0.41	−0.18	2.25	2.25
BB3	−1.07	−0.16	2.23	2.24
M1	−0.78	0.17	2.27	1.83
M2	−0.79	0.18	2.27	1.87
M3	−0.86	0.19	2.27	1.97
M4	−0.70	0.17	2.27	1.87
M5	−0.79	0.19	2.27	2.08
M6	−0.75	0.18	2.27	1.86
*a*-TiO_2_				
top BB1	−2.60	0.03	1.93	2.25
top BB2	−3.56	0.00	1.94	2.24
top BB3	−2.96	−0.03	1.93	2.27
top M1	−2.20	0.33	1.96	1.60
top M2	−2.40	0.35	1.95	1.60
top M3	−2.38	0.35	1.95	1.63
top M4	−0.97	0.18	2.26	1.63
top M5	−0.80	0.16	2.26	1.62
bottom BB1	−4.43	0.04	1.91	1.95
bottom BB2	−4.92	0.08	1.97	2.00
bottom BB3	−0.28	−0.07	2.21	2.22
bottom M1	−1.18	0.18	2.25	1.69
bottom M2	−0.84	0.14	2.25	1.63
bottom M3	−1.53	0.19	2.23	1.62
bottom M4	−1.83	0.14	1.93	1.77

Rutile (110): Two bidentate (BB and M2B) and a monodentate configuration (M1) are identified. These are shown in Figure 3, and their adsorption energies are listed in Table 1. In the bidentate BB configuration, both oxygen atoms of the carboxylic group bind to surface Ti atoms with both O_mol_-Ti bonds equal to 2.21 Å. The H atom is dissociated from the molecule and is bound to a surface oxygen. In the monodentate configuration (M1), the molecule is undissociated, with O_mol_-Ti bonds of 2.26 Å and hydrogen bonds between the H atom and a surface oxygen of 1.60 Å. The configuration M2B was attempted as a monodentate configuration, where the molecular hydrogen coordinates to an in-plane oxygen atom of TiO_2_. This configuration, however, converged to a bidentate configuration in which AcOH preserves the H atom and in which a hydrogen atom of the CH_3_ group is 2.21 Å away from a surface O atom. This configuration is less stable than the dissociated bidentate configuration by more than 1 eV and has larger O_mol_-Ti bond lengths (2.27 and 2.29 Å) than the dissociated configuration. Similar to anatase, molecular adsorption has no appreciable effect on the band structure regardless of the configuration (Figure 1). The energetic preference of AcOH for bidentate configurations computed here (Table 1) is in agreement with known preference for bidentate bridging adsorption on the clean (110) surface identified experimentally [88,89].

(B) (001): Three bidentate (BB1 to BB3) and six monodentate configurations (M1 to M6) were identified and are shown in Figure 4. Their adsorption energies are listed in Table 1. BB1 and BB2 configurations, while visually similar, differ in that the COO group straddles different types of surface oxygen atoms, located in a slightly more “sagged” or slightly more protruding oxygen rows, respectively. This corresponds to significantly different *E_ads_* (−0.74 and −0.41 eV, respectively). The strongest bound configuration (*E_ads_* = −1.11 eV) is BB3, which straddles different Ti rows. In the BB configurations, both oxygen atoms of the carboxylic group bind to surface Ti atoms with both O_mol_-Ti bond lengths within 2.23–2.25 Å, with the shortest lengths corresponding to the strongest-bound BB3 configuration. In all monodentate configurations, the O_mol_-Ti bond length is 2.27 Å; the H_mol_-O_surf_ bonds are spread within 1.86–2.08 Å. Similar to anatase and rutile, molecular adsorption has no appreciable effect on the band structure regardless of the configuration (Figure 1; not all M configurations are shown to prevent clutter, but no configuration changed the DOS).

**Figure 5:**
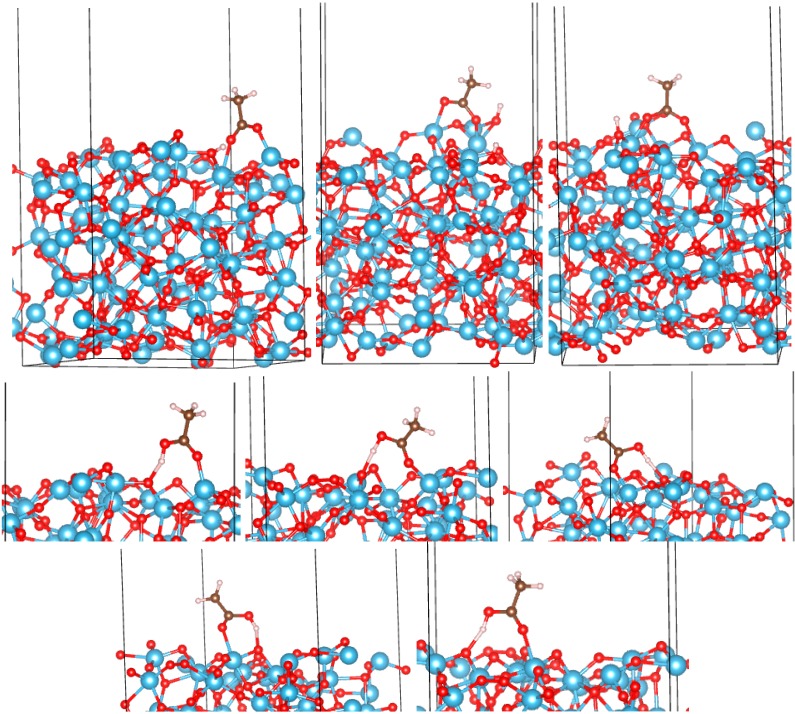
Adsorption configurations of AcOH on the “top” surface of *a*-TiO_2_. Top row: BB1 to BB3 (left to right). Bottom rows: M1 to M5 (left to right and top to bottom).

**Figure 6:**
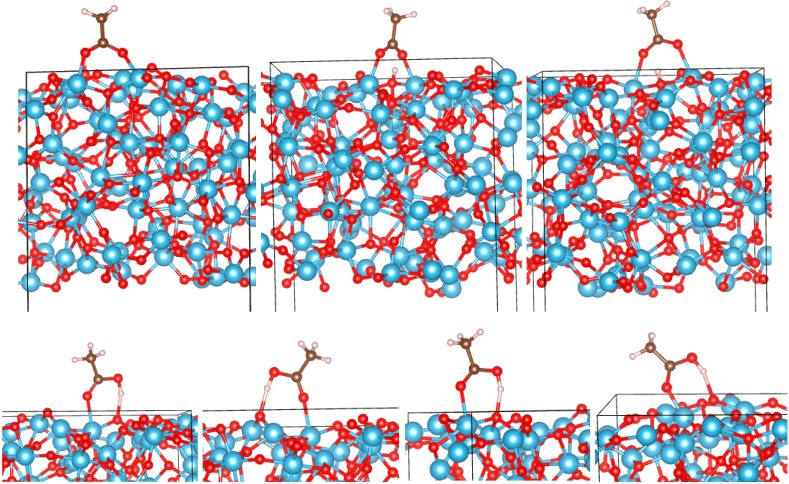
Adsorption configurations of AcOH on the “bottom” surface of *a*-TiO_2_. Top row: BB1 to BB3 (left to right). Bottom row: M1 to M4 (left to right).

*a*-TiO_2_: On both surfaces of *a*-TiO_2_ (“top” and “bottom”), we have identified a total of six bidentate and nine monodentate configurations. They are shown in Figure 5 and Figure 6, and their adsorption energies are listed in Table 1. While it is possible that other adsorption configurations exist on these surfaces, the 15 that we identified already provide an understanding of the strength of binding and the spread of adsorption energies on different sites of amorphous TiO_2_. Similar to the crystalline surfaces, bidentate binding is energetically preferred. The O_mol_-Ti bonds in BB configurations are generally shorter than on crystalline surfaces, the shortest reaching 1.91 Å (Table 1). While there is a dispersion of *E_ads_* values among both monodentate and bidentate configurations, the strongest *E_ads_* is larger than that of any crystalline surface in either the monodentate (reaching −1.83 eV) or bidentate (reaching −4.92 eV) regimes. Amorphous TiO_2_ can therefore be used to achieve stable anchoring of organic molecules, such as dyes, that bind via a -COOH group.

### 3.3. Correlates of Adsorption Strength

**Figure 7:**
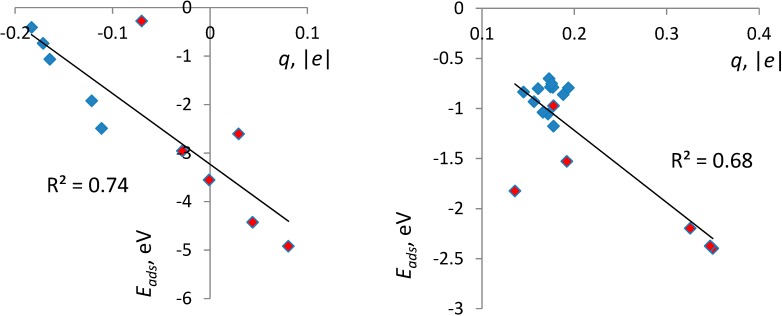
Dependence of the adsorption energy *E_ads_* on the molecular charge *q* in bidentate (left) and monodentate (right) configurations on all surfaces and their linear regressions. Points corresponding to *a*-TiO_2_ are highlighted in red.

On all surfaces, bidentate configurations are more strongly bound to the surface than monodentate, and from Table 1, it can be seen that increased binding strength generally corresponds to shorter molecule-surface distances and more positive charge on the molecule. In Figure 7, we quantify this dependence by plotting *E_ads_* *vs.* the molecular charge *q*. Points corresponding to adsorption on amorphous surfaces are highlighted. There is a significant linear trend, with only two outlier points for bidentate and one outlier for monodentate configurations, with all outliers corresponding to *a*-TiO_2_. This is not surprising given the expected differences of the chemical environments of the adsorbed molecule in different parts of an amorphous surface. Even so, the correlation is significant, with Pearson correlation coefficients (*R^2^*) of 0.69 and 0.88 for bidentate and monodentate configurations, respectively. Figure 7 also highlights that *E_ads_* in excess of −3 eV is only achieved in bidentate modes on *a*-TiO_2_ and only with positive *q* values, in contrast with bidentate binding on crystalline surfaces, where the molecule accepts charge (we note that the scales of *q* should not be directly compared for BB and M configurations, as in one case, H is dissociated and in the other, it is not; see Figure 2, Figure 3, Figure 4, Figure 5 and Figure 6).

Trends can also be identified between the adsorption energy and the shorter O_mol_-Ti distance, with correlation coefficients (*R^2^*) of 0.69 and 0.88 for bidentate and monodentate configurations, respectively. A combined linear regression in these two variables (*i.e*., charge and bond length) unveils a significant correlation with the correlation coefficient of 0.75 and 0.93 for bidentate and monodentate configurations, respectively.

## 4. Conclusions

We have presented the first comparative density functional tight binding study of acetic acid adsorption on anatase (101), rutile (110), B (001) and amorphous surfaces of TiO_2_. On all surfaces, bidentate bridging adsorption configurations are preferred, consistent with available data. The computed adsorption energies of the lowest-energy bidentate configurations *E_ads_* = −1.93, −2.49 and −1.09 eV on anatase, rutile and (B)-TiO_2_, respectively. For monodentate configurations, the strongest *E_ads_* = −1.06, −1.11 and −0.86 eV on anatase, rutile and (B)-TiO_2_, respectively. The comparison with previously reported DFT values for *E_ads_* computed with different computational setups highlights the need to use one and the same setup to obtain truly comparative values of *E_ads_* among different phases and configurations.

Multiple monodentate and bidentate configurations are identified on *a*-TiO_2_ with a distribution of adsorption energies and with the lowest binding energy stronger than that of the crystalline counterparts, with *E_ads_* up to −4.92 eV for bidentate and −1.83 eV for monodentate adsorption. Amorphous TiO_2_ can therefore be used to achieve strong anchoring of organic molecules, such as dyes, that bind via a -COOH group. While the presence of the surface leads to a contraction of the band gap *vs.* bulk, molecular adsorption caused no appreciable effect on the band structure around the gap in any of the systems.

Then, comparison among five surfaces (three crystalline and two amorphous) and a total of 30 binding geometries and energies allowed us to identify correlations between the binding strength, the charge donation at the interface and bond lengths between molecular and surface atoms. There is a strong positive correlation between the adsorption strength and the positive charge on the molecule, as well as between *E_ads_* and the shortest bond length between molecular O and surface Ti atoms.
